# Induction of Reactive Bone Stromal Fibroblasts in 3D Models of Prostate Cancer Bone Metastases

**DOI:** 10.3390/biology12060861

**Published:** 2023-06-15

**Authors:** Louisa C. E. Windus, Nicholas Matigian, Vicky M. Avery

**Affiliations:** 1 Discovery Biology, Centre for Cellular Phenomics, Griffith University, Nathan, QLD 4111, Australia; 2 QCIF Facility for Advanced Bioinformatics, Institute for Molecular Bioscience, The University of Queensland, Brisbane, QLD 4072, Australia; n.matigian@qcif.edu.au; 3 School of Environment and Science, Griffith Sciences, Griffith University, Nathan, QLD 4111, Australia

**Keywords:** prostate cancer, tumour–stromal microenvironment, reactive bone stromal fibroblasts, 3D cultures, EMT/MET

## Abstract

**Simple Summary:**

When prostate cancer cells spread to other parts of the body (metastasise), they frequently migrate to the bone. Here they encounter a microenvironment that differs greatly from that in the primary tumour (prostate), namely interactions with bone-specific cells, including fibroblasts. To better understand the contribution of these fibroblasts to tumour progression, it is the aim of the current paper to propagate a biologically relevant fibroblast population that mimics the cellular and molecular profile of fibroblasts as found in the metastatic bone environment in the body. Using three-dimensional (3D) cell culture models, we were able to transform normal fibroblast cells into fibroblasts that express modified protein and gene profiles similar to those found in fibroblasts in the metastatic bone environment in the body. The exploitation of these engineered 3D models could help further unravel the novel biology regulating metastatic growth and the role fibroblasts play in the colonisation process. Elucidation of these processes has the potential to aid in the development of therapies not currently available for late-stage cancers.

**Abstract:**

A dynamic interplay between prostate cancer (PCa) cells and reactive bone stroma modulates the growth of metastases within the bone microenvironment. Of the stromal cells, metastasis-associated fibroblasts (MAFs) are known to contribute but are the least studied cell type in PCa tumour progression. It is the aim of the current study to establish a biologically relevant 3D in vitro model that mimics the cellular and molecular profiles of MAFs found in vivo. Using 3D in vitro cell culture models, the bone-derived fibroblast cell line, HS-5, was treated with conditioned media from metastatic-derived PCa cell lines, PC3 and MDA-PCa 2b, or mouse-derived fibroblasts 3T3. Two corresponding reactive cell lines were propagated: HS5-PC3 and HS5-MDA, and evaluated for alterations in morphology, phenotype, cellular behaviour, plus protein and genomic profiles. HS5-PC3 and HS5-MDA displayed distinct alterations in expression levels of N-Cadherin, non-functional E-Cadherin, alpha-smooth muscle actin (α-SMA), Tenascin C, and vimentin, along with transforming growth factor receptor expression (TGF β R1 and R2), consistent with subpopulations of MAFs reported in vivo. Transcriptomic analysis revealed a reversion of HS5-PC3 towards a metastatic phenotype with an upregulation in pathways known to regulate cancer invasion, proliferation, and angiogenesis. The exploitation of these engineered 3D models could help further unravel the novel biology regulating metastatic growth and the role fibroblasts play in the colonisation process.

## 1. Introduction

Bone metastasis is a frequent occurrence in late-stage prostate cancer (PCa) patients and represents more than 90% of PCa-related deaths [1]. Primary and metastatic PCa progression has long been recognised as the product of a complex cross-talk between different cell types within the tumour and its surrounding supporting tissue, the tumour–stroma [2,3]. Of these stromal cells, cancer-associated fibroblasts (CAFs) have been identified as a crucial mediator of tumour progression at the primary site [4]. However, when PCa cells metastasise to the red bone marrow, they encounter a microenvironment that differs greatly from that in the primary tumour, with the inclusion of interactions with bone-specific cells such as osteoblasts, osteoclasts, and bone-derived fibroblasts. These tumour–stroma interactions are often exquisitely tissue-specific, and processes that are relevant to primary cancer might not be reflected in the metastatic environment. Although it has been reported that metastatic cells arrive at the bone together with stromal cells from the primary tumour [5], the cancer cells need to instruct the new environment to form the stroma necessary to support colonisation and growth. Stromal fibroblasts at metastatic sites are termed metastasis-associated fibroblasts (MAFs). To date, the role MAFs play in this colonisation process within the metastatic bone niche is largely unknown in PCa, and studies examining MAF–tumour interactions are rare. Thus, to better understand the contribution of different tumour microenvironment (TME) components in metastatic PCa progression, it is the aim of the current paper to propagate a biologically relevant bone-derived fibroblast population that mimics the cellular and molecular profile of MAFs found in vivo.

Of the studies undertaken in PCa, it has been established that MAFs are closely involved in the formation of metastatic bone lesions [6,7,8], and mesenchymal stem cells (MSCs) from bone may possibly be the progenitor of MAFs in metastatic bone tumours [8]. MAFs have been shown to have similar antigenic profiles and expression of signalling molecules to that of CAFs, including upregulated alpha-smooth muscle actin (α-SMA), fibroblast growth factor (FGF), Wnt, epidermal growth factor (EGF), and a decrease in transforming growth factor (TGF-β) signalling in patient-derived tissue and mouse xenograft models [6,9,10]. The activation of these signalling pathways by MAFs promotes the development of bone metastases via stimulating metastatic tumour cells within the bone microenvironment to secrete factors that result in the osteolytic destruction of bone [11]. Others have implicated deregulation of interleukin (IL)-1β receptor (IL-1R) [8] and heparan sulfate proteoglycan (HSPG2) [12] in MAF signalling, favouring and supporting PCa colonisation within the bone. A more complete picture of the role MAFs play during the colonisation process comes from studies involving other cancers, including lung and breast. In these studies, MAFs are the main component of the tumour stroma and can remodel the extracellular matrix (ECM) by expressing factors such as fibronectin, TGFβR2 [13], collagen 1 [14], and matrix metalloproteinase-2 (MMP2) [15]. Strategies targeting MAFs themselves for the treatment of metastatic cancer are limited to preclinical models [16]. Further elucidation of the cellular, molecular, and genomic regulation of these very important cells is warranted and could lead to the development of much-needed late-stage cancer treatments.

Earlier PCa studies established that the growth of both benign and malignant prostate tumours in vivo was dependent on the stromal cells of the host environment, and the enhanced tumorigenicity was the result of changes in the genotype and phenotype of both the prostate cancer cells and the host stromal cells [17,18]. Moreover, stromal cells that have been treated with conditioned media taken from tumour cells or have been extracted after cell–cell contact with tumour cells retain the ability to transform non-tumourigenic cells, indicating that the phenotypic and genotypic alterations of the stromal cells were permanent [19]. Bone marrow stromal cells, particularly HS5 cells, have been used in vitro to mimic the bone marrow microenvironment and have the ability to strongly promote prostate cancer cell invasion in both metastatic PCa cell lines (PC3) and non-tumourigenic (LNCaP) cells [20]. More recently, HS-5 cell lines have been reported to reproduce not only the bone marrow MSC capacity to influence tumour biology but to evaluate the molecular mechanisms underlying tumour immune escape mediated by stroma cells [21]. For these reasons, HS5 cells were used in the current study. To investigate the potential of invasive and non-invasive bone-derived PCa cells in transforming non-tumourigenic cells to a MAF profile, two representative cell lines of PCa bone metastasis, PC3 and MDA-PCa-2b, were evaluated in this study. PC3 cells are characterised as invasive and castrate-resistant, while MDA-PCa-2b are androgen-sensitive and less invasive in nature [22]. Mouse embryonic fibroblast cell lines (3T3) have been used for over three decades as feeder cells in human keratinocyte cultures to produce cultured epidermal autografts for the treatment of burns and wounds and have been used extensively in corneal cell therapies [23,24,25]. The cross-species protein interactions between 3T3 and human cells have been well established [26,27]. The secretome of 3T3 cells has been shown to promote unrestricted cell proliferation, reprogramming, and immortalisation of human cells [27], processes that have been implicated in tumourigenesis. To investigate whether the secretome of these cells could induce a reactive MAF signature, 3T3 cells were also utilised in the current study.

Epithelial-to-mesenchymal transition (EMT) and its counter phenomenon referred to as mesenchymal-to-epithelial transition (MET) have been suggested to play crucial roles in metastatic dissemination and seeding to distant sites in a range of cancers [28,29]. EMT processes are proposed to provide cancer cells with the ability to detach from the primary tumour, break down the basement membrane and invade either locally or distantly [30]. Upon seeding at a new site, metastatic EMT cells are hypothesised to revert to an epithelial state via MET [31]. To date, studies investigating EMT/MET mediation within the bone niche have been undertaken primarily in tumour cells, not the surrounding stroma. Irregular expression of EMT markers, including E-cadherin, platelet-derived growth factor D (PDGF-D), Notch-1, nuclear factor kappa-light-chain-enhancer of activated B cells (NF-κB), vimentin, and zinc finger E-box binding homeobox 1 (ZEB-1) have been observed with Notch-1 expression considered the signature for the acquirement of EMT in PCa bone metastasis [32,33,34]. However, other studies have reported metastatic colonisation in PCa models to be MET independent [35]. Investigation of EMT/MET signatures of MAFs is, to date, lacking in the current literature and is an aim of the current paper.

Here we evaluate and compare protein and genomic signatures of two reactive-induced bone stromal cell lines, HS5-PC3 and HS5-MDA, using three-dimensional (3D) in vitro models. In comparison to untreated HS5 (control) or HS5-3T3 treated cells, reactive HS5-PC3 and HS5-MDA cells presented with alterations in morphology, proliferation, and expression of EMT/MET markers, and reactive stromal markers, including vimentin, E-Cadherin, N-Cadherin, TGF-β RI, TGF-β R2, tenascin C, and α-SMA. Our results suggest that while the reactive cell lines display different EMT/MET antigenic profiles, both exhibited changes in TGF-β R2 receptors accompanied by an upregulation of Tenascin C expression that mimics, more precisely, the profiles of MAFs found in vivo. Moreover, transcriptomic analysis revealed a shift in genetic profiles of reactive HS5-PC3 cells towards a tumorigenic signature with a clear upregulation in pathways known to mediate cancer invasion, proliferation, and angiogenesis. Therefore, these reactive cell lines may serve as a valid and biologically representative model for the study of stromal interactions in mediating metastatic PCa progression.

## 2. Materials and Methods

### 2.1. PCa Cell Lines

Cell lines were purchased commercially and authenticated by ATCC using cytochrome c oxidase subunit I (COI) gene analysis for interspecies identification and STR analysis (DNA profiling) for intra-species identification. Verification of these cell lines was undertaken by ATCC only; no further authentication processes were undertaken after purchase from ATCC. Mycoplasma testing was routinely undertaken on the cell lines used in this study. The PCa cell lines (MDA-PCa 2b and PC3), mouse-derived 3T3 fibroblast, and bone-stromal-derived fibroblasts cell line (HS5) were cultured in RPMI-1640 (Sigma–Aldrich) + 10% foetal bovine serum (FBS, Gibco). MDA-PCa 2b, PC3, HS5, and 3T3 cells were grown in cell-cultured-treated T75 Flasks (Falcon). All cells were maintained in standard culture conditions (5% CO_2_ at 37 °C). Media was replenished every two days. All assays in this paper used cells with low passage numbers < 10–12.

### 2.2. Propagation of Reactive HS5 Cell Lines HS5-PC3, R-HS5-MDA, and HS5-3T3

All reactive HS5 cell lines were propagated on cells with small passage numbers (<10–12), and the total time for establishing reactive cells was six days in culture. To induce mitotic inactivation in 3T3 cells, cells were grown to 95–100% confluency for up to two days, and conditioned media (CM) was collected. The CM from PC3 and MDA-PCa 2b cells were collected from cells when they reached 50–60% confluency and collected every two days for up to six days or until cells reached 80% confluency. The CM was centrifuged at 300× *g* for 5 min, filtered through a 0.22 μm filter, and stored at −80 °C until use. CM was then directly transferred to HS5 cells after 24 h initial seeding in T75 flasks. CM was replaced every three days, and HS5 cells were treated for six days. During these six days, cells that had reached 80–90% confluency were replated (2/10) in T75 flasks, and after 4 h initial seeding, CM was reinstated. After six days, CM was removed, and HS5 cells were then maintained in RPMI-1640, supplemented with 10% foetal bovine serum for two passages before freezing down aliquots and storing them in liquid nitrogen. Two reactive cell lines were propagated: HS5-PC3 and HS5-MDA. Fresh aliquots of each reactive cell line were then propagated and maintained in standard cell culture conditions as outlined previously. Control HS5 (non-treated) cells were simply maintained in RPMI-1640, supplemented with 10% foetal bovine serum.

### 2.3. 3D Cultures

Miniaturised 3D cultures were propagated using an established protocol [36,37]. Briefly, 45 µL growth factor-reduced (GFR) Matrigel™/culture medium (70%: BD Biosciences) was added to 96-well plates and polymerised at 37 °C with 5% CO_2_ for 1 h. Cultures of treated fibroblasts HS5-PC3, HS5-MDA, HS5-3T3, and untreated HS5 cells were seeded at ~800 cells per well and maintained in standard culture conditions. Media was removed and replenished every three days. Cultures were maintained for up to nine days.

### 2.4. 3D Bulk Cultures for Protein Extraction

Protein extraction was obtained from 3D cultures grown in 12-well plates with 450 µL GFR Matrigel™/culture medium (70%: BD Biosciences) added per well and polymerise at 37 °C with 5% CO_2_ for 1 h. Single-cell cultures were seeded at ~8000 cells per well, and media was replaced every three days. Following six and nine days in culture, 3D cultures were extracted using Cell Recovery Solution (CRS: BD Biosciences). The culture medium was removed, washed twice (cold PBS), and 1 mL of cold CRS was applied. The cells and matrix were transferred into 15 mL tubes using a wide bore 1 mL pipette tip and incubated at 4 °C on a rocker for 1.5 h. Cells were then centrifuged (800× *g* for 10 min) at 4 °C, washed with cold PBS, and further centrifuged (500× *g* for 7 min). Cell pellets were lysed, and western blotting was performed.

### 2.5. Western Blotting

Cells were harvested from 3D cultures and lysed in cold RIPA buffer (75 mM TrisCl pH 8, 150 mM NaCl, 0.1% SDS, 1% Triton-X-100, 0.5% deoxycholic acid) containing protease inhibitors (Roche), incubated at 4 °C (30 min) and centrifuged at 14,100× *g* (20 min). Supernatants were assayed using an RCDC Protein Assay (BIO-RAD). Equal amounts of protein were loaded onto SDS-PAGE gels for electrophoresis and then transferred to Polyvinylidene Fluoride (PVDF) membranes in a transfer buffer (25 mM Tris Base, 200 mM glycine containing 15% methanol) for 30 min using a Bio-Rad Turbo-Blot. PVDF membranes were blocked (5% non-fat milk powder for 1 h) and washed with Tris-buffered saline with Tween 20 (TBST), and primary antibodies were applied overnight at 4 °C in a blocking buffer. These included anti-human N-cadherin (0.5 µg/mL, R&D Systems), goat anti-TGF βR1 (0.2 µg/mL; R&D Systems), goat anti-human vimentin (0.2 µg/mL, R&D Systems), goat anti-αSMA (2 µg/mL, Abcam), and goat anti-TGF βR2 (0.2 µg/mL; R&D Systems). Membranes were washed, and HRP-conjugated secondary antibodies (Bio-Rad) were applied for 1 h at room temperature. Membranes were washed and imaged on a Versa Doc (Bio-Rad) gel imager. Membranes were stripped and re-probed for β-actin. Image Lab software was used to undertake densitometric analysis and was expressed as a fold change normalised against β-actin. Photoshop was used to crop full-length blots without further non-linear digital enhancement. Full-length blots are available in Supplementary Materials.

### 2.6. Immunocytochemistry

Miniaturised 3D cultures of reactive and non-reactive HS5 cells were washed (3 × 5 min PBS) and fixed with 4% PFA for 20 min. For immunofluorescence labelling, cells were washed (3 × 5 min PBS), permeabilised, and blocked overnight (O/N) with 2% BSA, 0.1% Triton-X, 0.05% TWEEN20 at 4 °C. Cells were further washed (1 × 5 min PBS/0.1%TX, 2 × 5 min PBS) and the following primary antibodies were applied O/N at 4 °C in a blocking buffer: mouse anti-E-cadherin (2 µg/mL, Invitrogen), goat anti-human vimentin (0.2 µg/mL, R&D Systems), goat anti-TGF βR1 (0.2 µg/mL; R&D Systems), goat anti-TGF βR2 (0.2 µg/mL; R&D Systems), anti-human N-cadherin (0.5 µg/mL, R&D Systems), goat anti-αSMA (2 µg/mL, Abcam), and mouse anti-tenascin C (0.5 µg/mL, R&D Systems). Cells were washed with PBS (3 × 5 min), incubated with appropriate secondary antibodies (5 µg/mL 488 goat anti-mouse/rabbit anti-goat, 5 µg/mL 594, goat anti-mouse/rabbit anti-goat), nuclear stain Hoechst (1/1000, Invitrogen), and/or filamentous actin stains, Texas Red Phalloidin (1/80, Invitrogen), for 4 h at room temperature (R/T). Cells were washed and then imaged using the PerkinElmer Opera™ Confocal Imager and an Olympus IX-81 Scanning Confocal microscope.

### 2.7. Proliferation Assays

To assess cell proliferation, assays were performed using an established protocol described elsewhere [36,37].

### 2.8. Migration Assays

Reactive and non-reactive HS5 cells were plated in 12-well corning plates at ~10,000 cells per well and left to adhere overnight in normal culture conditions. A yellow pipette tip was then used to scratch the confluent monolayers. The media were replaced with fresh medium, and the scratch area was analysed after 6, 10, and 18 h using images obtained on an InCell 2000 Imager (General Electric, Boston, MA, USA) and processed by the measurement tool in AxioVision 4.8 software (Carl Zeiss, Oberkochen, Germany).

### 2.9. Live and Fixed Cell Imaging

All fixed cells were imaged using either an Olympus IX-81 Scanning Confocal microscope, with an Olympus PlanNeo-FLUOR 40/1 oil iris, or a PerkinElmer Opera™ Quadruple Excitation High Sensitivity Confocal Cell Imager with a PerkinElmer 20/0.75 water iris. Live cell imaging was completed on the InCell 2000 (GE) Cell Imager using a GE 20/0.75 air iris. Images were created using Adobe Photoshop CS4 without further non-linear digital manipulation.

### 2.10. 3D Bulk Cultures and RNA Extraction for Microarray Analysis

RNA extraction for microarray analysis was obtained from 3D Matrigel cultures grown in 12-well plates. For 3D cultures, 450 µL GFR Matrigel™/culture medium (70%: BD Biosciences) was added per well and allowed to polymerise at 37 °C with 5% CO_2_ for 1 h. Single-cell cultures were then seeded at ~8000 cells per well, and the media was replenished every three days. Single-cell populations HS5 and PC3 were seeded at ~4000 cells per well in co-culture (HS5 + PC3). After six days in culture, 3D bulk cultures of HS5 (Control), PC3, HS5-PC3, and PC3 were treated with HS5 CM, and co-cultures of HS5/PC3 were extracted using Cell Recovery Solution (CRS: BD Biosciences) as outlined in section: 3D bulk cultures for protein extraction. RNA was isolated using QIAGEN RNeasy Mini Kit and quantified using a Nanodrop ND1000 and an Agilent 2100 Bioanalyser for RNA integrity. Four biological repeats of each cell culture type were collected and ran on an Illumina microarray bead plate.

### 2.11. Microarray Procedures and Analysis

The RNA samples were amplified and hybridized to human whole-genome Illumina Human-Refseq8 v2 BeadChips (Illumina, Inc. San Diego, CA, USA). Slides were scanned on an Illumina Beadstation and analysed using BeadStudio Version 3.1.7 (Illumina, Inc. San Diego, CA, USA). Initially, genes were filtered for reliably expressed probes. For a probe to be included in downstream analysis, it had to be detected (Illumina detection score > 0.99) in each replicate of its group. Differential expression (DE) analysis was performed with the LIMMA package in R [38,39]. The *p*-values were adjusted for multiple comparisons with the Benjamini–Hochberg correction method. Significance was set at (*p* ≤ 0.001). Further analysis of differentially expressed gene sets was undertaken using Gostat (IPA; 0.6 Agilent Technologies), and FEN diagrams were established using Adobe Illustrator CS4. For canonical pathway analysis, disease, and function, the −log (*p*-value) 1.3 was taken as a threshold, the Z-score > 2 was defined as the threshold of significant activation, while Z-score < −2 was defined as the threshold of significant inhibition.

### 2.12. Quantification Procedures and Statistical Analysis

An Envision plate reader was employed to quantify proliferation rates using fluorescence excitation/emission settings of 530 nm/595 nm. Mean fluorescence readings were then normalised and imported into Graph Pad Version 5 for further statistical analysis. The mean spheroid area was quantified using the outline measurement tool in AxioVision Rel 4.8 software (Carl Zeiss, Boston, MA, USA). A minimum of *n* = 10 images were acquired for each variable using ×10 bright field magnification on an Olympus BX150. Mean intensity values for antigenic profiles were calculated from a minimum of *n* = 15–20 images per variable and processed using the surfaces analysis tool available in Imaris x64 software (Bitplane, Belfast, UK). All statistical analyses were carried out using Graph-Pad Prism (Version 5), and statistical significance for all given variables was tested using the Kruskal–Wallis test and Dunn’s multiple comparison test for post hoc analysis. The significance level was set at *p* < 0.05

## 3. Results

### 3.1. Characterisation of Reactive HS5 Cell Line Morphology and Phenotypic Appearance in 3D Matrices

**Figure 1:**
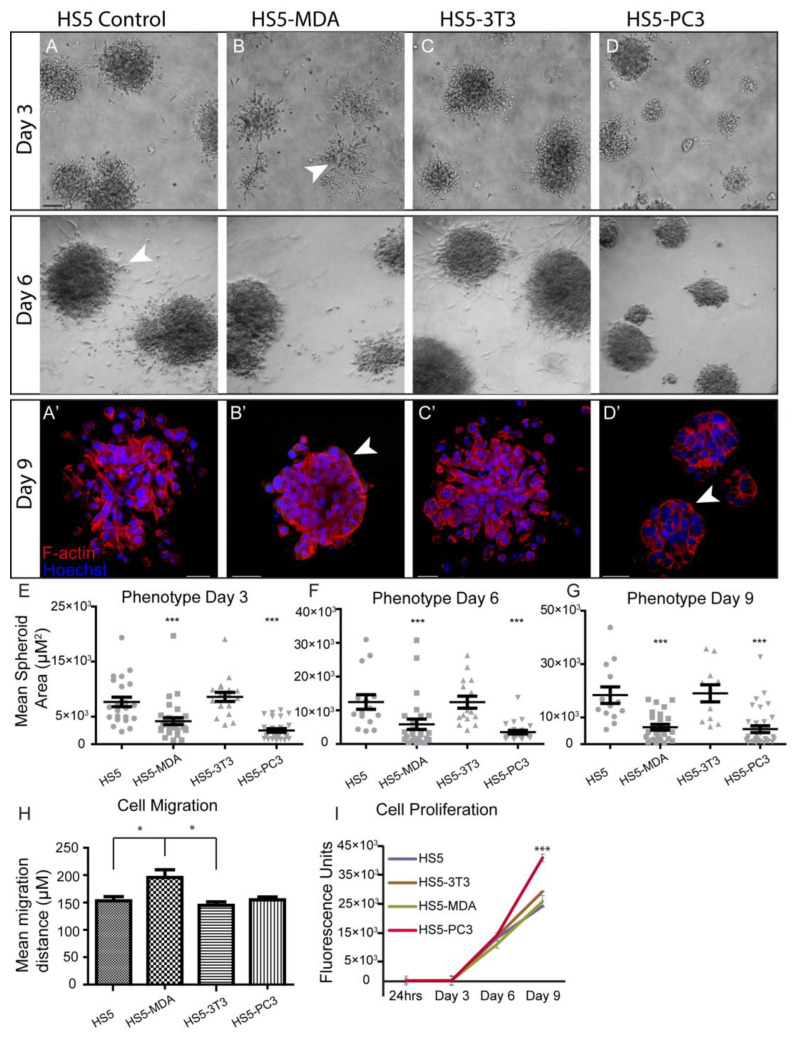
Alteration in cellular morphology, behaviour, migration, and proliferation in HS5-MDA and HS5-PC3 cell lines in 3D monocultures. (**A**) Differential Interference Contrast (DIC) images of non-reactive HS5 bone stromal cells on days three and six. (**A’**) F-actin staining of a non-reactive HS5 spheroid mass on day nine. (**B**) DIC images of reactive R-HS5-MDA cells on days three and six. (**B’**) F-actin staining of R-HS5-MDA spheroid mass on day nine showing the formation of basement membrane (arrow). (**C**) DIC images of R-HS5-3T3 cells on days three and six. (**C’**) F-actin staining of R-HS5-3T3 spheroid mass on day nine. (**D**) DIC images of R-HS5-PC3 cells on days three and six. (**D’**) F-actin staining of R-HS5-PC3 spheroid mass on day nine showing the formation of a basement membrane (arrow). (**E**–**G**) Graphs depicting the quantification of mean spheroid area of all cell lines plated in 3D across days three, six, and nine (*n* = 4). (**H**,**I**) Graphs depicting quantification of cellular migration after 18 hs (*n* = 5) (**H**), cellular proliferation on days three, six, and nine (*n =* 4) (**I**) (* *p* < 0.05, and *** *p* < 0.001). Error bars denote S.E.M. Scale bars = 30 μm.

To investigate differences in morphological characteristics and cell junction formation of HS5-MDA, HS5-PC3, HS5-3T3, and non-reactive HS5 cell lines (control) grown in 3D monocultures, we used brightfield optics and immunocytochemical staining procedures. When plated on the Matrigel matrix; all stromal cell lines clearly differentiated and formed relevant multi-cellular structures (Figure 1). Consistent with our previous findings [36], by day three in culture, non-reactive HS5 stromal cells formed rounded masses (Figure 1A), and by day six was marked by a meshwork of disorganised and loosely aggregated interlacing cells that formed extending processes into the matrix (Figure 1A; Day 6; arrowhead). Utilising F-actin staining, we could discern that, consistent with a mesenchymal phenotype, these masses clearly lacked an organised structure and acinar formation (Figure 1A’). Similarly, HS5 cells treated with conditioned media from murine 3T3 fibroblasts for over six days displayed a very similar phenotype to that of untreated HS5 cells (Figure 1C,C’).

When HS5 cells were treated for over six days with conditioned media taken from either MDA-PCa 2b cells or PC3 cells, these cells displayed a distinctly different phenotype from control cells. Across all days in culture, HS5-MDA and HS5-PC3 cells formed significantly smaller aggregates in comparison to non-treated HS5 cells and HS5-3T3 cells (Figure 1E–G). By day three, HS5-MDA formed irregular-shaped clusters with stellate radiating tubular processes, consistent with a metastatic cell line (Figure 1B; arrowhead). By day six, these cells showed a remarkable reversion of phenotype; they presented as well-organised and rounded spheroid structures (Figure 1B; Day 6). F-actin staining on day nine (Figure 1B’) revealed HS5-MDA cells presented as an ordered rounded collection of aggregates with well-organised cell–cell adhesions (Figure 1B’; arrowhead). Similarly, in comparison to untreated HS5 (control) and HS5-3T3 cells, HS5-PC3 cells formed rounded, more organised spheroids with little evidence of protruding cellular processes into the matrix (Figure 1D; Day 3 and 6). By day nine, F-actin staining revealed HS5-PC3 cells to aggregate into well-organised grape-like spheroid structures (Figure 1D’). These results suggest that both HS5-MDA and HS5-PC3 cells formed aggregates that demonstrated a partial reversion of phenotype consistent with an epithelial presentation, although no acinar formation was evident. These results also suggest that these phenotypic changes were PC3 and MDA-PCa 2b-specific, as HS5 cells grown in embryonic fibroblastic (3T3)-treated media were unchanged.

### 3.2. Alterations in Cellular Behaviour, Proliferation and Migration in Reactive Cell Lines

To investigate possible alterations in cellular behaviour, assays were undertaken to measure the proliferation and migration of HS5-MDA, HS5-PC3, HS5-3T3, and non-reactive HS5 (control) cell lines. Utilising the traditional wound migration assays, we found that HS5-MDA cells migrated at significantly higher rates over a 12 h period (Figure 1H), while HS5-PC3 cells exhibited upregulated proliferative (Figure 1I) properties. These results suggest that while reactive cell lines HS5-PC3 and HS5-MDA displayed similar alterations in morphology, their behavioural profiles differ.

### 3.3. Alterations in EMT Protein Profiles of Reactive HS5 Cell Lines

To further analyse possible differences in the expression and distribution of EMT/MET associated proteins, including E-Cadherin, N-Cadherin, and vimentin, immunocytochemical staining and mean fluorescence intensity analysis were undertaken together with total protein analysis in HS5-MDA, HS5-PC3, HS5-3T3, and non-reactive HS5 cell lines on days six and nine in 3D culture.

#### 3.3.1. Alterations in E-Cadherin Expression

**Figure 2:**
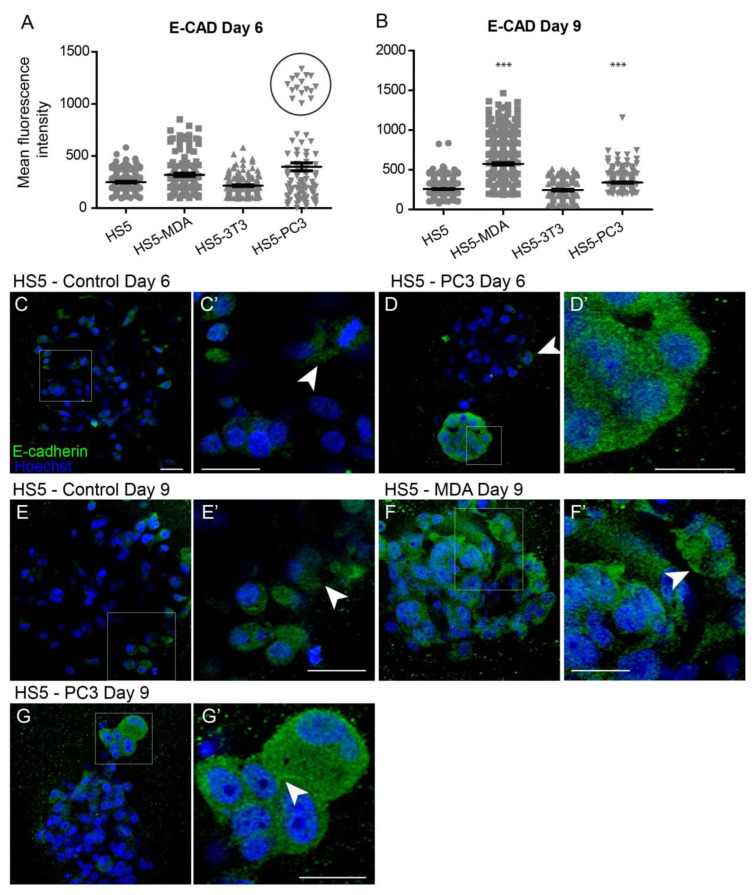
Alterations of E-Cadherin expression on HS5-MDA and HS5-PC3 cell lines in 3D culture. (**A**,**B**) Quantification of mean fluorescence intensity of E-Cadherin expression on all cell lines on day six (A) and day nine (B) (*n* = 5). (**C**–**G’**) Panels show immunocytochemical expression of E-Cadherin (green fluorescence). (**C**) E-Cadherin expression on non-reactive HS5 cells is primarily distributed in the cytoplasm (insert box; (**C’**) arrowhead). (**D**) E-Cadherin expression on reactive HS5-PC3 cells is upregulated in a population of cells (insert box) with the distribution primarily in the cytoplasm (**D’**). (**E**) E-Cadherin expression on non-reactive HS5 cells on day nine continues to be distributed in the cytoplasm (insert box; (**E’**) arrowhead). (**F**) By day six, HS5-MDA cells display an upregulation of E-Cadherin expression distributed in the cytoplasm of cells (Insert box, (**F’**)). (**G**) E-Cadherin expression on reactive HS5-PC3 cells on day nine is upregulated in a population of cells (insert box) with the distribution in the cytoplasm and membrane (insert box, (**G’**); arrowhead). (*** *p* < 0.001). Error bars denote S.E.M. Scale bars = 30 μm.

Quantification of the mean fluorescence intensity of E-Cadherin expression revealed no significant differences between all cell lines on day six (Figure 2A). Interestingly, however, the box plot revealed a subpopulation of HS5-PC3 cells that were found to express higher intensity values (Figure 2A; circle). Immunofluorescence staining of E-Cadherin revealed minimal presence of this protein on untreated HS5 cells (Figure 2C) with positive staining isolated to the cytoplasm (Figure 2C’; arrowhead), consistent with a non-functional protein. This distribution pattern was also observed in HS5-3T3, HS5-MDA cells, and HS5-PC3 cells on day six (Figure 2D; arrowhead). However, in ~25% of images, there were subpopulations of HS5-PC3 spheroids with a clearly upregulated cytoplasmic E-Cadherin expression (Figure 2D’).

By day nine, quantification of E-Cadherin fluorescence revealed a significant upregulation of intensity on both HS5-MDA and HS5-PC3 cells (Figure 2B). In comparison to untreated HS5 cells (Figure 2E,E’; arrowhead), E-Cadherin intensity and distribution were clearly altered in HS5-MDA (Figure 2F) and HS5-PC3 cells (Figure 2G), with the distribution apparent in the cytoplasm and rarely present on the membrane (Figure 2F’; arrowhead, Figure 2G’; arrowhead).

#### 3.3.2. Alterations in N-Cadherin Expression

**Figure 3:**
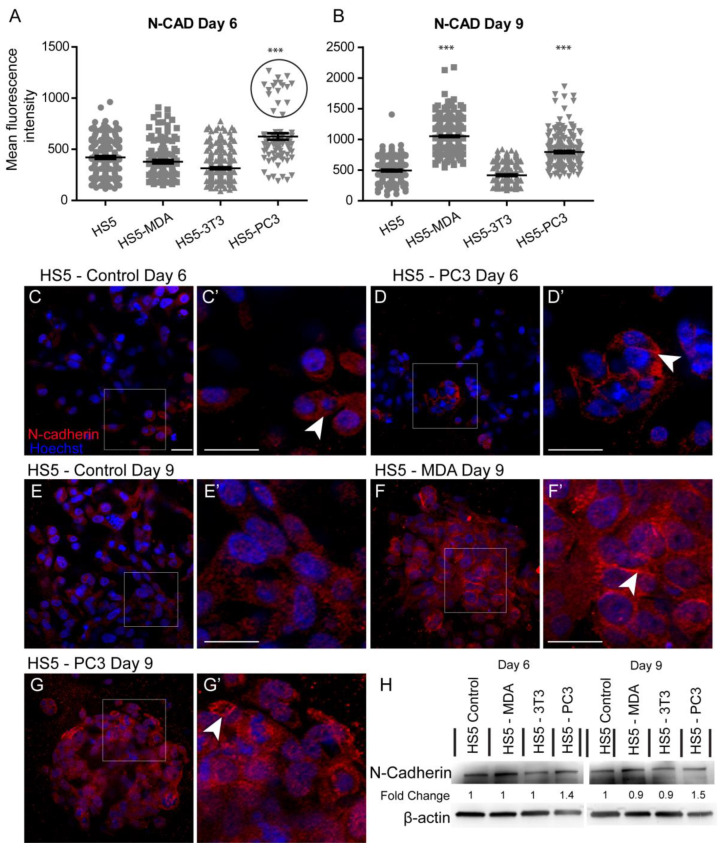
Alterations in N-Cadherin expression on HS5-MDA and HS5-PC3 cell lines in 3D culture. (**A**,**B**) Quantification of mean fluorescence intensity of N-Cadherin expression on all cell lines on day six (**A**) and day nine (**B**) (*n* = 5). (**C**–**G’**) Panels show immunocytochemical expression of N-Cadherin (red fluorescence). (**C**) Expression of N-Cadherin in non-reactive HS5 cells is primarily distributed in the cytoplasm (insert box; (**C’**) arrowhead). (**D**) N-Cadherin expression on reactive HS5-PC3 cells is upregulated in a population of cells (insert box) with the distribution primarily membrane-bound (insert box, (**D’**); arrowhead). (**E**) N-Cadherin continues to be primarily distributed in the cytoplasm (insert box; (**E’**) arrowhead) on non-reactive HS5 cells on day nine. (**F**) By day nine, N-Cadherin expression is found in both the cytoplasm and membrane of HS5-MDA cells (insert box, (**F’**) arrowhead). (**G**) N-Cadherin expression on reactive HS5-PC3 cells continues to be upregulated with the distribution in both the cytoplasm and membrane (insert box, (**G’**) arrowhead). (**H**) Western blot and densitometric analysis of N-Cadherin expression on all cell lines on day six and day nine. (*** *p* < 0.001). Error bars denote S.E.M. Scale bars = 30 μm. The uncropped western blot figures are presented in Figure S1.

Quantification of the mean fluorescence intensity of N-Cadherin expression revealed a significant increase between untreated HS5 and HS5-PC3 cells on day six (Figure 3A). Similar to results found for E-Cadherin, a subpopulation of HS5-PC3 cells was found to express higher intensity values (Figure 3A; circle). Immunostaining of N-Cadherin expression on HS5 cells (Figure 3C) revealed positive staining isolated to the cytoplasm (Figure 3C’; arrowhead), consistent with a non-functional protein. This distribution pattern was also observed on HS5-3T3 with similar total protein present to that of untreated HS5 cells (Figure 3H). N-Cadherin expression in HS5-PC3 cells revealed a membrane-bound distribution (Figure 3D’), indicative of a functional protein. Densitometry revealed a 1.4-fold increase in HS5-PC3 cells on day six (Figure 2H and Figure S1). By day nine, quantification of N-Cadherin fluorescence intensity revealed a significant upregulation on both HS5-MDA and HS5-PC3 cells (Figure 3B). In comparison to untreated HS5 cells whose distribution remained in the cytoplasm (Figure 3E,E’), N-Cadherin distribution was apparent in the cytoplasm and the membrane of HS5-MDA cells (Figure 3F,F’; arrowhead) and HS5-PC3 cells (Figure 3G,G’; arrowhead). Densitometry results confirmed an up-regulation of N-Cadherin with a 1.5-fold difference in total protein on HS5-PC3 cells on day nine (Figure 3H and Figure S1).

#### 3.3.3. Alterations in Vimentin Expression

**Figure 4:**
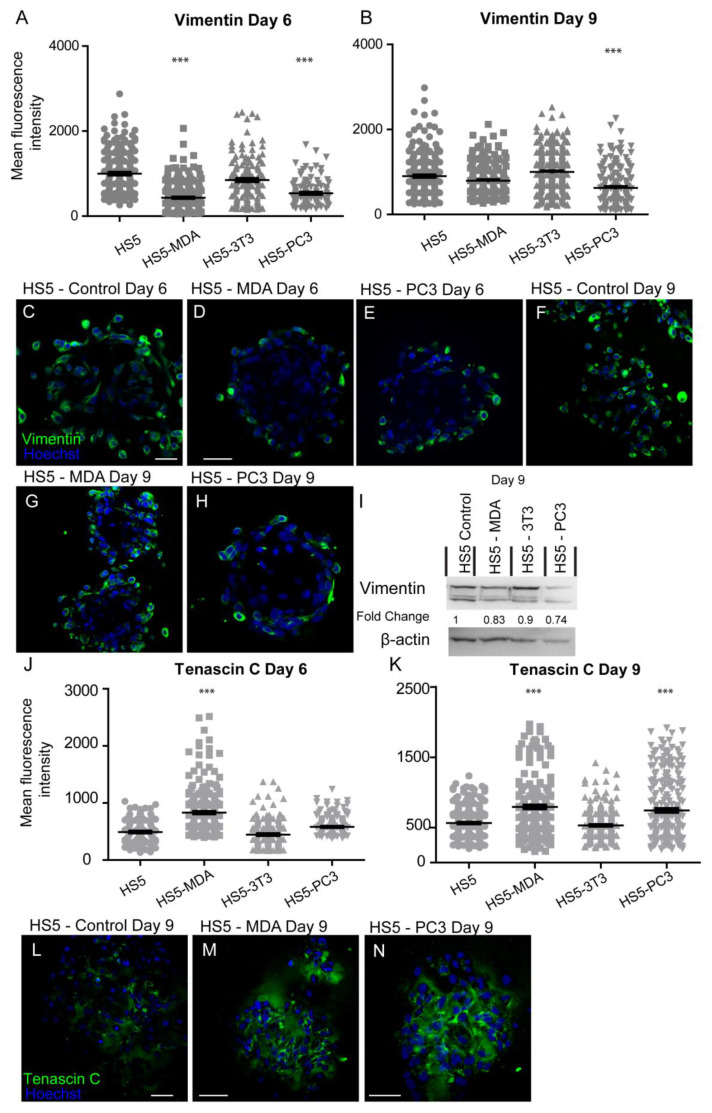
Expression of vimentin and tenascin C on HS5-MDA, HS5-PC3, HS5-3T3, and non-reactive HS5 cell lines after six and nine days in 3D culture. (**A**,**B**) Quantification of mean fluorescence intensity of vimentin expression on all cell lines on day six (**A**) and day nine (**B**) (*n* = 5). Panels show immunocytochemical expression of vimentin and tenascin C (green fluorescence). Vimentin expression on HS5 control (**C**) HS5-MDA (**D**) and HS5-PC3 cells (**E**) on day six and vimentin expression on HS5 control (**F**) HS5-MDA (**G**) and HS5-PC3 cells (**H**) on day nine. (**I**) Western blot and densitometric analysis of vimentin expression on all cell lines on days six and nine. The uncropped western blot figures are presented in Figure S1. (**J**,**K**) Quantification of mean fluorescence intensity of tenascin C expression on all cell lines on day six (**J**) and day nine (**K**) *(n* = 5). Tenascin C expression on HS5 control (**L**), HS5-MDA (**M**), and HS5-PC3 cells (**N**) on day nine. (*** *p* < 0.001). Error bars denote S.E.M. Scale bars = 30 μm.

After six days in culture, the intensity of vimentin expression was found to be significantly decreased on both HS5-MDA and HS5-PC3 cells when compared to untreated HS5 and HS5-3T3 cells (Figure 4A). Immunocytochemical staining supported these findings, with both the presence and distribution of vimentin clearly diminished on both HS5-MDA (Figure 4D) and HS5-PC3 cells (Figure 4E) when compared to untreated HS5 cells (Figure 4C) or HS5-3T3 cells. By day nine, however, the intensity of vimentin was found to be significantly decreased in HS5-PC3 cells, while HS5-MDA cells retained similar intensity values to those found with untreated HS5 and HS5-3T3 cells (Figure 4B). Results obtained via western blotting revealed a decrease in total protein expression in both HS5-PC3 and HS5-MDA cells, with a 0.74- and 0.83-fold difference reported, respectively (Figure 4I and Figure S1).

Taken together, these results suggest that HS5-MDA and HS5-PC3 cells clearly displayed alterations in proteins associated with EMT/MET expression profiles. HS5-PC3 cells showed upregulation of non-functional E-Cadherin and functional N-Cadherin with concomitant loss of vimentin expression, indicative of a MET programme. A similar expression pattern was demonstrated by HS5-MDA cells but did not retain changes in N-Cadherin, indicative of a partial conversion to MET.

### 3.4. Expression Profiles of HS5-MDA and HS5-PC3 Cells Are Consistent with Reactive Protein Signatures as Found In Vivo

After six days in culture, the intensity of tenascin C protein was found to have significantly increased on HS5-MDA when compared to untreated HS5 and HS5-3T3 cells (Figure 4J). By day nine, however, both HS5-MDA and HS5-PC3 cells displayed a clear upregulation in intensity (Figure 4K). The distribution of tenascin C was found throughout the ECM as a fibrous network interlaced through cells, with some distribution apparent on the membrane, consistent with its enormously diverse range of functions. In comparison to untreated cells (Figure 4L), tenascin C presence and distribution were more evident within the ECM and membrane of reactive HS5 cell lines (Figure 4M,N).

**Figure 5:**
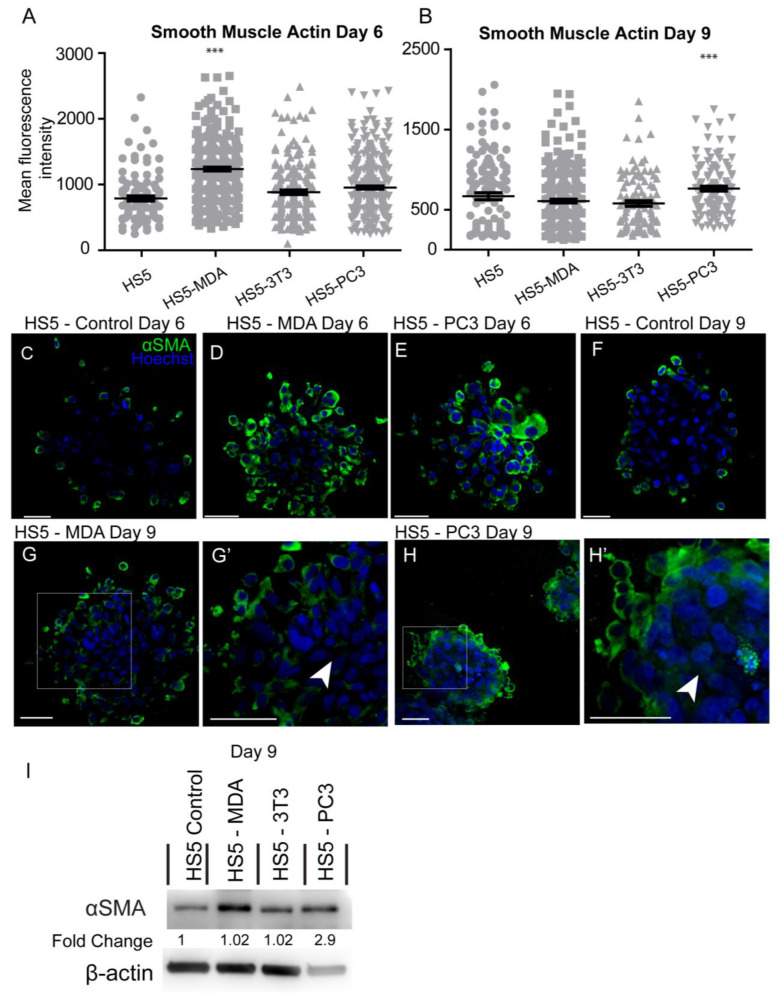
Alteration in the expression of smooth muscle actin (αSMA) on HS5-MDA and HS5-PC3 cell lines in 3D culture. (**A**,**B**) Quantification of mean fluorescence intensity of αSMA expression on all cell lines on day six (**A**) and day nine (**B**) (*n* = 5). Panels show immunocytochemical expression of αSMA (green fluorescence). αSMA expression on HS5 control (**C**), HS5-MDA (**D**), and HS5-PC3 cells (**E**) on day six and αSMA expression on HS5 control (**F**) on day nine. αSMA expression of HS5-MDA (**G**,**G’**) and HS5-PC3 cells (**H**,**H’**) on day nine. (**I**) Western blot and densitometric analysis of αSMA expression on all cell lines on day nine. (*** *p* < 0.001). Error bars denote S.E.M. Scale bars = 30 μm. The uncropped western blot figures are presented in Figure S1.

Investigation of the mean intensity of alpha-smooth muscle actin (αSMA) protein on day six in culture revealed significant increases in HS5-MDA cells when compared to untreated HS5 cells (Figure 5A). By day nine, the mean intensity was found to be significantly elevated in HS5-PC3 cells only (Figure 5B). Immunocytochemical staining revealed that in comparison to untreated HS5 cells on day six or day nine (Figure 5C,F, respectively) or HS5-3T3 cells (results not shown), both HS5-MDA (Figure 5D,G,G’; arrowhead) and HS5-PC3 cells (Figure 5E,H,H’ arrowhead) clearly illustrating a greater and more uniform distribution of the protein throughout the spheroid mass. Western blotting results and densitometric analysis confirmed an increase in total protein levels on day nine with a fold change of 2.9 on HS5-PC3 cells only (Figure 5I and Figure S1).

**Figure 6:**
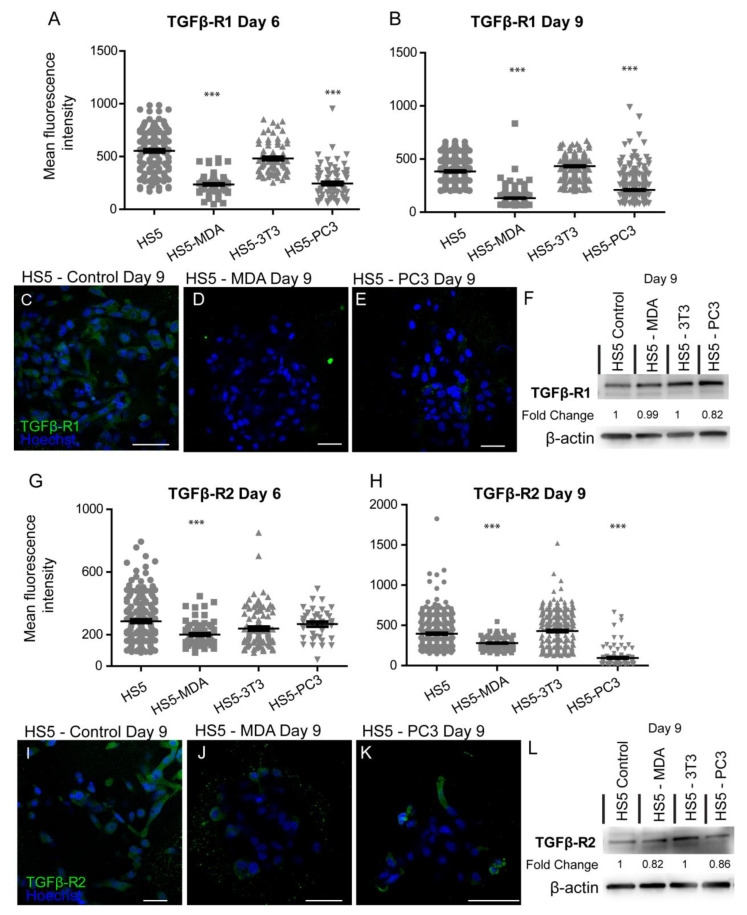
Alteration in the expression of TGF-βR1 and βR2 on HS5-MDA and HS5-PC3 cell lines in 3D culture. Quantification of mean fluorescence intensity of TGF-βR1 expression on all cell lines on day six (**A**) and day nine (**B**) (*n* = 5). Panels show immunocytochemical expression of TGF-βR1 (green fluorescence) on HS5 control (**C**), HS5-MDA (**D**), and HS5-PC3 (**E**) cells on day nine. (**F**) Western blot and densitometric analysis of TGF-βR1 expression on all cell lines on day nine. Quantification of mean fluorescence intensity of TGF-βR2 expression on all cell lines on day six. The uncropped western blot figures were presented in Figure S1. (**G**) and day nine (**H**) (*n* = 5). Panels show immunocytochemical expression of TGF-βR2 (green fluorescence) on HS5 control (**I**), HS5-MDA (**J**), and HS5-PC3 (**K**) cells on day nine. (**L**) Western blot and densitometric analysis of TGF-βR2 expression on all cell lines on day nine. (*** *p* < 0.001). Error bars denote S.E.M. Scale bars = 30 μm. The uncropped western blot figures are presented in Figure S1.

A loss of TGFβ-R1 expression was reported for both reactive stromal cell lines. Mean immunohistochemistry intensity results confirmed these findings at both day six (Figure 6A) and day nine (Figure 6B), with a decrease in the distribution of these receptors on both HS5-MDA (Figure 6D) and HS5-PC3 cells (Figure 6E) in comparison to their non-treated counterparts (Figure 6C). Western blotting results and densitometric analysis confirmed a reduction in total protein level with a fold change of 0.82 for HS5-PC3 cells only (Figure 6F and Figure S1). Immunofluorescence intensity results for TGFβ-R2 confirmed HS5-MDA cells displayed consistent decreases in protein expression across days six and nine in culture (Figure 6G,H,J), while HS5-PC3 showed a significant decrease in protein by day nine (Figure 6H,K). These results were confirmed via western blot (Figure 6L and Figure S1) with a recorded 0.82-fold decrease in HS5-MDA cells and a 0.86-fold decrease in HS5-PC3 cells on day nine.

### 3.5. Transcriptomic Expression Patterns of Reactive Stromal Cell Lines

**Figure 7:**
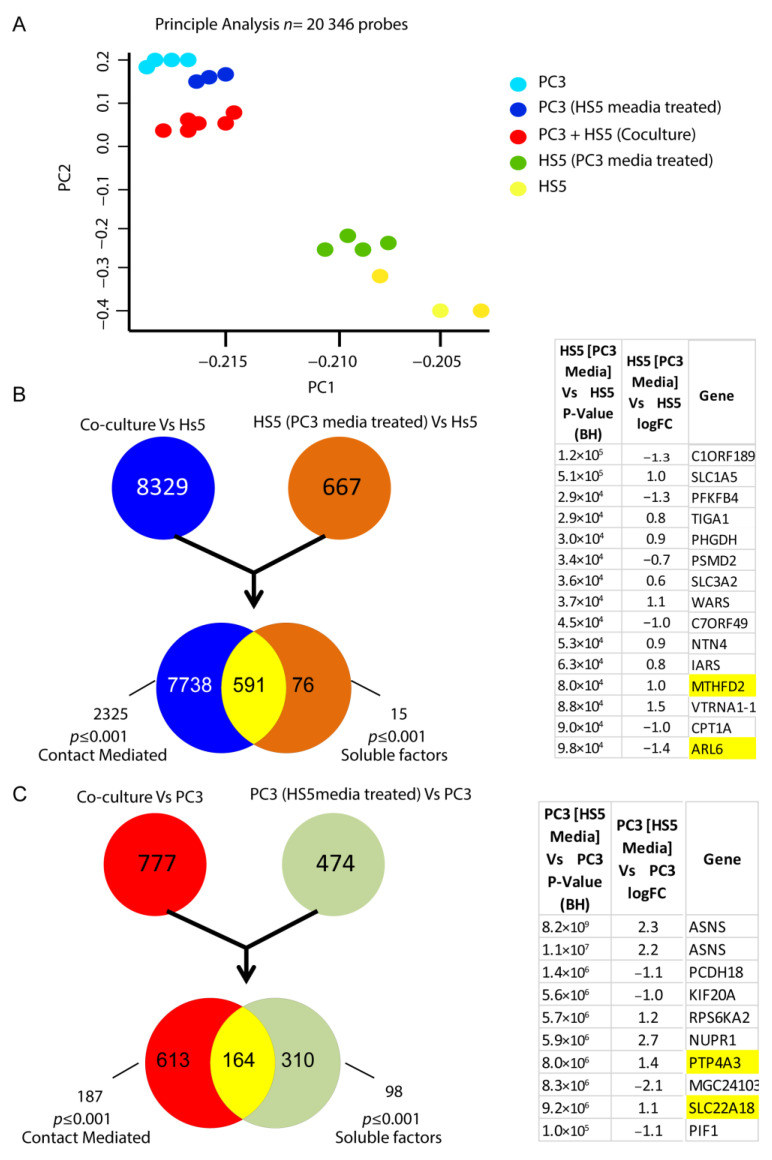
Alteration in the transcriptomic signatures of HS5-PC3 cells (**A**) Microarray principal analysis of HS5 control (yellow *n* = 3), PC3 (blue *n* = 4), HS5-PC3 (green *n* = 4), co-culture (PC3 + HS5: red *n* = 6) and PC3 cells conditioned with HS5 media (purple *n* = 3). (**B**) FEN diagram outlining 15 gene alterations in HS5-PC3 cells. (**C**) FEN diagram outlining alterations in over 98 genes when PC3 cells are treated with HS5 conditioned media, with the 10 most significant gene alterations listed. Genes highlighted in yellow have been reported previously in other cancer progressions and/or reported as potential pharmaceutical targets in advanced cancers.

Initial principal analysis taken from the microarray assay revealed group-specific differential transcript signatures when compared across 23,000 transcript variants for each cell-derived group (Figure 7A). Although distinct in their subsets, when HS-5 cells were co-cultured with PC3 cells (direct cell–cell contact), the variation in gene signature indicated a shift toward a metastatic genotype reminiscent of PC3 cells alone (Figure 7A). Similarly, when cultured with CM from PC3 cells, HS5 cells exhibited a distinct shift in genomic signatures in comparison to untreated HS5 cells (Figure 7A). Transcriptomic analysis of these CM-treated HS5 cells revealed alterations in 15 genes when compared to their non-treated counterparts, which are listed in Figure 7B. Additionally, alterations in over 98 genes were observed when PC3 cells were treated with non-reactive HS5 media in culture, with 10 of the most significant gene differentiations listed in Figure 7C.

**Figure 8:**
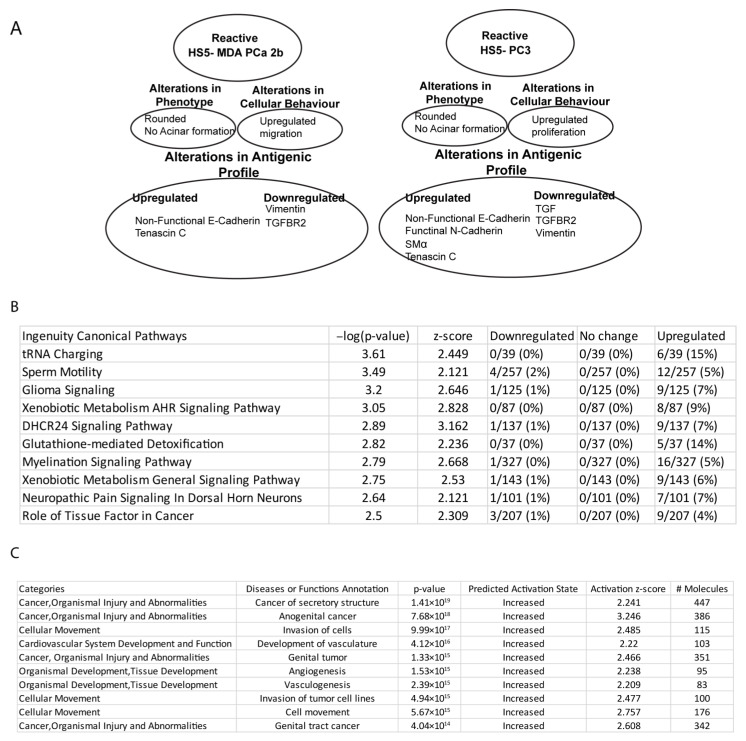
Summary profiles of HS5-PC3 and HS5-MDA cells and pathway analysis (IPA). (**A**) Summary profiles of reactive HS5 cell lines, HS5-MDA, and HS5-PC3. (**B**) Canonical pathway analysis of HS5-PC3 cells with the 10 most significant categories and function/disease annotations listed. (**C**) Gene ontology analysis of HS5-PC3 cells with the 10 most significant categories and function annotations listed.

Canonical pathway analysis of PC3-HS5 cells revealed dysregulation in over 30 pathways (Table S1), with the 10 most significant listed in Figure 8B. These were found to be upregulated and include dysfunction in tRNA and metabolic function. For a full list of associated molecules, please see Table S1. Gene ontology analysis of HS5 PC3 cells (Figure 8C) revealed significant alterations in over 50 pathways, with the 10 most significant listed in Figure 8C. These were predicted to be increased and involved transcripts that regulate cancer-mediated invasion, proliferation, angiogenesis, and vasculogenesis. For a full list of associated molecules, please see Table S1.

## 4. Discussion

Our results suggest that after treatment with media collected from prostate cancer cell lines, PC3 and MDA-PCa 2b, we were able to induce cellular, protein, and genomic changes to the bone-derived stromal cell line, HS5. Of interest, we have propagated two cell lines that express different EMT/MET signatures with the HS5-PC3 cell line maintaining a myofibroblastic phenotype, consistent with MAF populations reported in metastatic clinical tissue [40,41]. The differences in antigenic and cellular profiles between the two reactive cell lines are summarised in Figure 8A. It has been established that fibroblast populations are heterogenous across the bone marrow environment with distinct genomic profiles [42]. To this end, we propose that a combination of both reactive types and/or selection of subpopulations of cells could be a valid in vitro model to further evaluate the novel biology that regulates metastatic dissemination in Pca.

In vitro studies have shown that progenitors of MAFs become activated when treated with a conditioned medium from tumour cells [13,43,44]. Of the myriad of soluble factors identified from the secretome of cancer cells, ligands belonging to the TGF-β superfamily [45,46], platelet-derived growth factors (PDGFs) [47], inflammatory cytokines, and chemokines such as interleukins (IL) [48], and more recently, exosomes and macrovesicles [49] have been implicated in this transformative process. While both PC3 and MDA-Pca 2b cells were originally isolated from bone metastases, their secretome profiles are diverse. PC3 cells express high levels of TGF-β1 & β2, PDGF subunits A & B, IL-1β, and PC3-secreted microprotein (PSMP) [50,51,52]. Alternatively, MDA-Pca 2b cells secrete high levels of growth/differentiation factor-15 (GDF 15) and fibronectin and low levels of TGF-β1 [50,52]. These secretome factors have been implicated in having different roles in tumourigenesis, disease progression, prognosis, clinical outcome, and responses to chemo- and radiotherapy [53,54]. It is likely that the resultant differences seen in the cellular and antigenic profiles of these two reactive cell lines can largely be attributed to the presence or absence of these soluble factors, particularly the presence of TGF-β.

Consistent with the findings of others [6], our immunohistochemical and protein results suggest that TGF-β R2 is downregulated in both reactive cell lines, which infers reduced TGF-β signalling due to attenuated ligand binding. Down-regulation in TGF β signalling via receptor attenuation in both malignant and bone stromal cells has been shown to increase tumour growth and invasion [55]. The precise mechanisms mediating this process have not yet been elucidated. However, canonical pathway analysis of TGF-β signalling in HS5-PC3 cells revealed that of the 96 genes, 5 were listed as altered but did not reach significance indicating that TGF β signalling was not dysregulated (Table S1). Further studies are needed to verify the role TGF-β signalling plays in MAF formation and cross-talk in the colonisation process. Reduced TGF-β signalling activity has been reported to result in the upregulation of the activity of matrix metalloproteases particularly matrix metalloproteinase-2 and 9 (MMP-2 and MMP-9) in prostate epithelial cells [56]. Whether these same processes are evident in the bone microenvironment needs verification. Interestingly, both canonical and gene ontology analysis indicated enhanced MMP12 expression in HS5-PC3 cells (Table S1), which is consistent with previous research outcomes implicating MMP12 with castration-resistant prostate cancer (CRPC) progression and tumour metastasis [57]. MMP12 has been reported as a promising therapeutic target to treat intracranial tumours [58] and may be a potential target for late-stage metastases.

Transcriptomic analysis of the HS5-PC3 cell line revealed alterations in over 667 transcripts (Figure 7B). To evaluate the effects of soluble factors, data-based filtering revealed 15 transcripts of interest (Figure 7B). Of these alterations, ADP-ribosylation factor-like 6 (ARL6), transcript variant 1 is known to mediate the protein conducting channel Sec61β, and downregulation of this channel has been shown to increase resistance to known chemotherapeutic agents in ovarian cancer cells [59]. Similarly, methenyltetrahydrofolate cyclohydrolase (MTHFD2) has recently been identified as a novel modulator of the EMT marker vimentin [60] and reported to be a potential pharmaceutical target in advanced cancers [61]. Canonical pathway analysis revealed that HS5-PC3 cells displayed deregulation of many genes related to amino acid metabolism, including amino acid uptake, synthesis, and tRNAs charging (Figure 8B). Of interest was the upregulation of aminoacyl-tRNA synthetases (AARS), including methionyl-tRNA (MARS1) and leucyl-tRNA (LARS1). Upregulation of both these AARSs has been implicated in mediating poor clinical outcomes in breast cancer, bile duct adenocarcinoma, and non-small-cell lung carcinoma (NSCLC) and correlates with increased cell proliferation, migration and EMT [62,63,64]. More recently, it has been suggested that the catalytic domains of MARS1 have the potential as a therapeutic target [65]. Similarly, LARS1 has been reported to have a crucial role in the activation of mTORC1 and may present as a novel link between synthetase activity and tumourigenesis [66]. The role these AARS may play in mediating colonisation and metastatic progression in PCa is unknown and warrants further investigation.

Gene ontology analysis of HS5-PC3 cells revealed a common pattern of dysregulated genes known to mediate processes associated with cancer invasion, proliferation, angiogenesis, and vasculogenesis (Figure 8C), particularly at the primary site of tumour progression. CD44, caveolin-1, interleukin-1 receptor-like 1 (IL1RL1), integrin subunit alpha 5 (ITGA5), tumor necrosis factor-alpha (TNF-alpha), and mothers against decapentaplegic homolog 3 (SMAD3) were amongst the 30+ genes consistently reported across the functional categories listed in Figure 8C. Each of these genes has been reported widely in mediating cancer progression; however, their roles in MAF formation and colonisation are not well characterised. Noteworthy was the consistent upregulation of SMAD3 in HS5-PC3 cells. Considering TGF-β signalling was not transcriptionally enriched in HS5-PC3 cells, it is likely that in this model, activin/nodal signalling may regulate these processes. How SMAD/Activin signalling may mediate cross-talk in this TME is the work of future studies. The presence or absence of TGF-β may account for the differences in migratory and proliferative profiles between HS5 MDA and HS5-PC3 cells. TGF-β can indirectly participate in apoptosis, epithelial-to-mesenchymal transition, migration, proliferation, differentiation, and matrix formation [67]. Alternatively, fibronectin, a major secretome of MDA PCa 2b cells, has been associated with alterations in cell migration and invasion [68]. Exposure to high concentrations of fibronectin has been shown to decrease cell-substrate attachment, enabling higher migration rates in culture [69]. Alternatively, TGF-β plays a dual role in prostate cancer; in the initial stages, it acts as an anti-proliferative factor, while in the advanced stages, it acquires pro-metastatic and pro-oncogenic qualities [70]. The mechanisms used by these secretome molecules and their associated downstream signalling pathways to mediate these changes in HS5 cells are the work of future studies.

A recent study has revealed the existence of five distinct fibroblastic clusters within the bone niche of mice, each with distinct gene expression signatures indicative of functional heterogeneity [42]. Fibroblast clusters 1 and 2 were reported to express progenitor and MSC markers indicating a possible niche that facilitate cancer growth and metastasis, while clusters 3–5 formed a continuum of transcription factors involved in bone-forming events [42]. Moreover, in an acute myeloid leukemia mouse model, changes in fibroblast clusters 2 and 5 were evident in comparison to normal bone marrow [42]. Investigation and systemic survey of changes in these fibroblast-based clusters in response to PCa progression and dissemination are yet to be published. Overall, these studies give credence to the idea that subpopulations of resident fibroblasts within the bone niche play a role in the colonisation process, and the activation of these subpopulations can give rise to MAFs with myofibroblastic signatures. How these cell populations then mediate the pre-metastatic niche to accommodate arriving cancer cells and/or maintain metastatic TME is still largely unknow in PCa.

In our hands, HS5-PC3 cells were found to have upregulated α-SMA and tenascin-C and down regulated vimentin, similar to MAF myofibroblastic phenotypes reported in metastatic models of PCa [9,10]. Moreover, decreased expression of vimentin has been shown to promote proliferation in cancer cells in vitro and in vivo [71] and corresponds to the increased proliferative profile of HS5-PC3 cells reported here. Of interest was the temporal regulation of these markers in HS5-MDA cells. Taken from day six in culture, these cells clearly expressed an upregulation of α-SMA and a concomitant downregulation of vimentin, consistent with a reactive myofibroblast phenotype. By day nine, however, this phenotype was not evident. The reasons for this reversion can, in part, be explained by the absence of IL-1β, TGF-β, and PDGF subunits in the MDA PCa 2b secretome. Fibroblasts are extremely resistant to stress-induced changes [72], and our results suggest that soluble factors such as fibronectin and growth/differentiation factor-15 (GDF 15) may induce a partial myofibroblastic signature consistent with a permanent change and upregulation in tenascin C, cytoplasmic E-Cadherin, and N-Cadherin.

Of interest was the upregulation of cytoplasmic E-Cadherin in both the reactive cell lines. Functional E-Cadherin is expressed and located at the cell membrane and is a calcium-dependent adhesive protein responsible for epithelial cell cohesion [73]. Loss of E-Cadherin expression or relocalisation of expression to the cytoplasm is associated with EMT and has been widely linked to a variety of cancer processes, including metastases [74]. Upregulation of this protein, albeit not functional, may help regulate alternate signalling pathways. Similar results have been recently reported from patient samples of metastatic colorectal cancer [73]. In these samples, cytoplasmic E-cadherin was strongly co-localised with vascular endothelial growth factor A (VEGF-A) staining and was found to be a predictor of disease outcome.

Noteworthy are the preliminary data concerning the treatment of PC3 cells with non-reactive HS5 media in culture. Our results suggest that soluble factors secreted by non-reactive stroma affect the immune response and tumour suppressor genes of PCa cells that further support the growth of the tumour. For example, expression and clinical significance of tumour suppressor gene solute carrier family 22 (organic cation transporter), member 18 (SLC22A18), in non-small cell lung cancer [75] and protein tyrosine phosphatase type IVA, member 3 (PTP4A3) gene in VEGF signalling and the process of pathological angiogenesis [76] has been reported previously. These results are indeed novel and now need further clarification in their potential role in mediating MAF–tumour interactions and as druggable targets for late-stage PCa.

## 5. Conclusions

To date, the published findings regarding the role bone-residing fibroblasts and resulting MAFs play in metastatic dissemination in PCa is scarce. Here we report two potential reactive cell lines that mimic MAFs reported in vivo. We propose that a synergistic effect of various subpopulations with differing signatures is needed to replicate, more precisely, the TME of the bone niche. Utilising these and other cell populations in a 3D in vitro model will potentially help elucidate the novel biologics that drive this unique microenvironment.

## Data Availability

Data are available upon request to Distinguished Vicky M Avery. Due to the confidential nature of work undertaken in this laboratory, data are not publicly available.

## Supplementary Materials

The following supporting information can be downloaded at: https://www.mdpi.com/article/10.3390/biology12060861/s1, Figure S1: Western blot images; Table S1: Canonical and ontology analysis.
