# Supplementary material for: Induction of Reactive Bone Stromal Fibroblasts in 3D Models of Prostate Cancer Bone Metastases
Source: Biology (Basel). 2023 Jun 15;12(6):861. doi: 10.3390/biology12060861 (PMC10294842; doi:10.3390/biology12060861)
Supplement: Supplementary file 1 [file biology-12-00861-s001.zip › biology-2398159-supplementary.pdf]

Supplementary Figure S1:

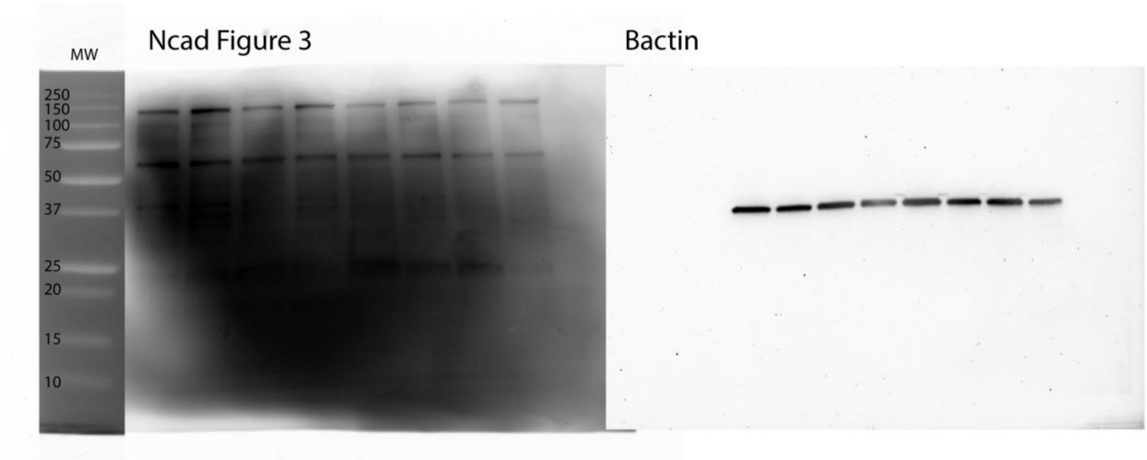

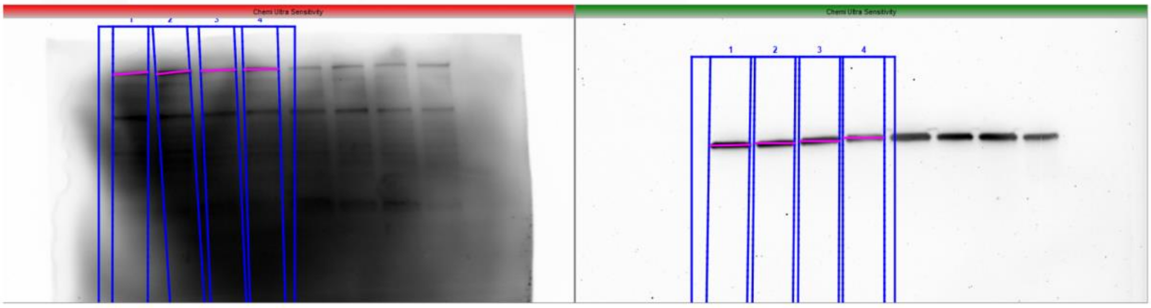

Lane	Band No.	Band Labe	Mol. Wt. (	Relative F	Adj. Volur	Volume (I	Abs. Quan	Rel. Quan	Band %	Lane %	Norm. Fac	Norm. Vol
1	1		N/A	0.140351	345746	7608826	N/A	N/A	100	18.84684	1	345746
1	1		N/A	0.318182	2178852	8665449	N/A	N/A	100	91.50888	1	2178852
2	1		N/A	0.133333	515658	7034610	N/A	N/A	100	20.68491	0.965325	497777
2	1		N/A	0.318182	2241603	8723910	N/A	N/A	100	90.87991	0.965325	2163876
3	1		N/A	0.129825	855576	7763492	N/A	N/A	100	31.24953	0.931239	796745
3	1		N/A	0.318182	2358720	9195342	N/A	N/A	100	92.25137	0.931239	2196531
4	1		N/A	0.129825	732156	7033478	N/A	N/A	100	35.02944	1.51921	1112298
4	1		N/A	0.318182	1402320	7023720	N/A	N/A	100	89.47476	1.51921	2130419

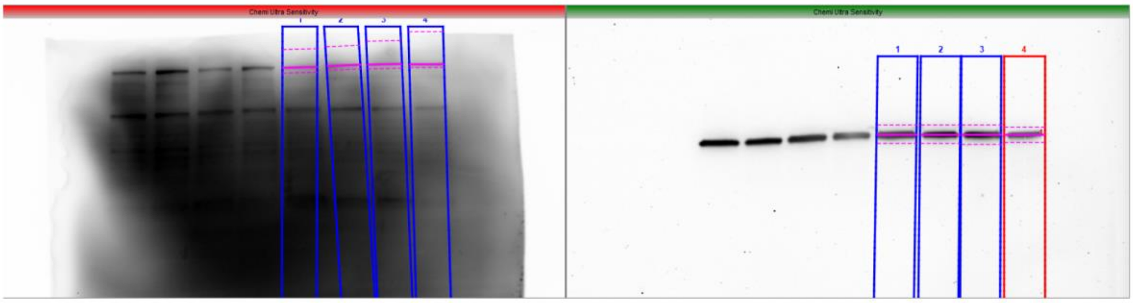

Lane	Band No.	Band Labe	Mol. Wt. (	Relative F	Adj. Volur	Volume (I	Abs. Quan	Rel. Quan	Band %	Lane %	Norm. Fac	Norm. Vol
1	1		N/A	0.157895	720392	5095104	N/A	N/A	100	9.553439	1	720392
1	1		N/A	0.35124	2414646	8175258	N/A	N/A	100	92.76201	1	2414646
2	1		N/A	0.154386	1926221	9167559	N/A	N/A	100	37.45267	1.013548	1952316
2	1		N/A	0.342975	2259220	8579704	N/A	N/A	100	87.96692	1.013548	2289827
3	1		N/A	0.147368	515644	4160920	N/A	N/A	100	12.23222	1.088399	561226
3	1		N/A	0.334711	2164773	8242494	N/A	N/A	100	90.51432	1.088399	2356137
4	1		N/A	0.14386	968558	5513270	N/A	N/A	100	31.47457	1.547448	1498793
4	1		N/A	0.322314	1501400	7426880	N/A	N/A	100	89.25429	1.547448	2323338

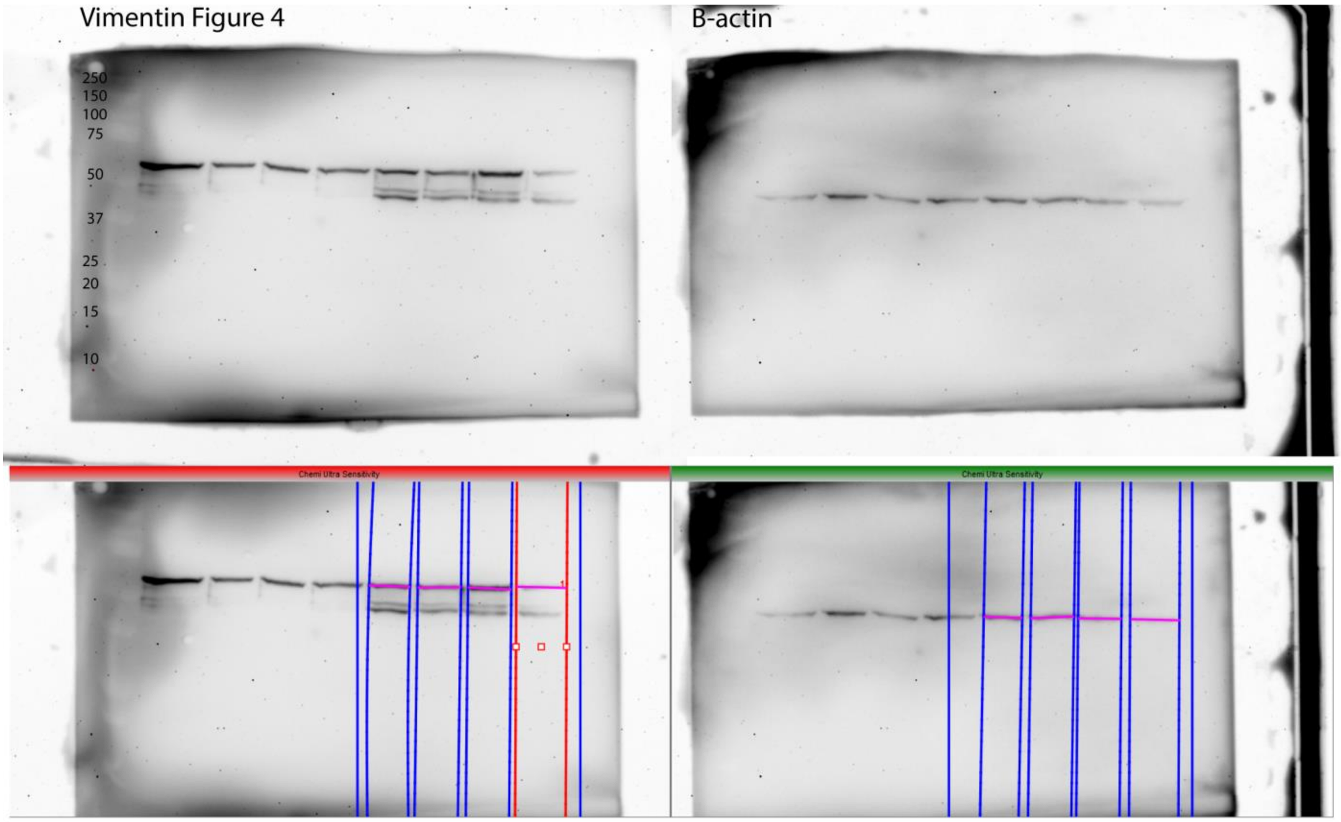

Lane	Band No.	Band Labe	Mol. Wt. (	Relative F	Adj. Volur	Volume (I	Abs. Quan	Rel. Quan	Band %	Lane %	Norm. Fac	Norm. Vol
1	1		N/A	0.355301	1503310	4915380	N/A	N/A	100	18.84087	1	1503310
1	1		N/A	0.429799	397344	1683712	N/A	N/A	100	12.23639	1	397344
2	1		N/A	0.358166	1260576	4514400	N/A	N/A	100	18.67361	0.830832	1047326
2	1		N/A	0.429799	437148	1863828	N/A	N/A	100	11.1848	0.830832	363196
3	1		N/A	0.361032	2017584	5742864	N/A	N/A	100	21.70868	0.957181	1931192
3	1		N/A	0.432665	255060	1491948	N/A	N/A	100	7.518358	0.957181	244138
4	1		N/A	0.358166	484386	2774856	N/A	N/A	100	9.057567	0.742568	359689
4	1		N/A	0.43553	169084	1239061	N/A	N/A	100	3.866564	0.742568	125556

3

4

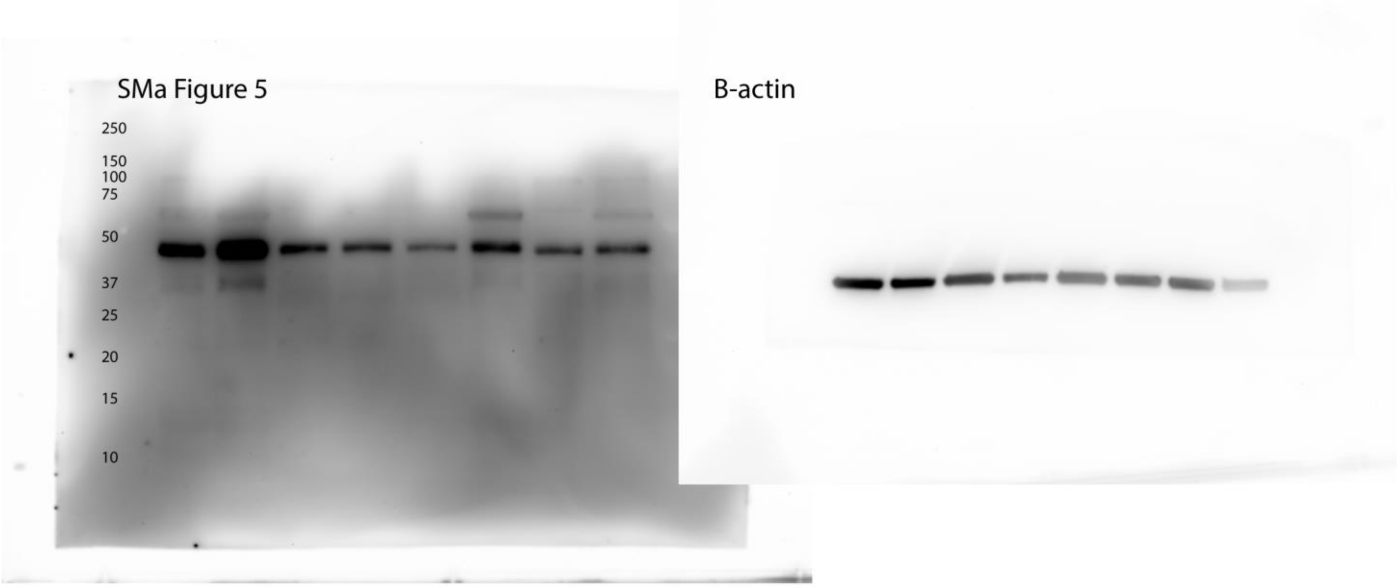

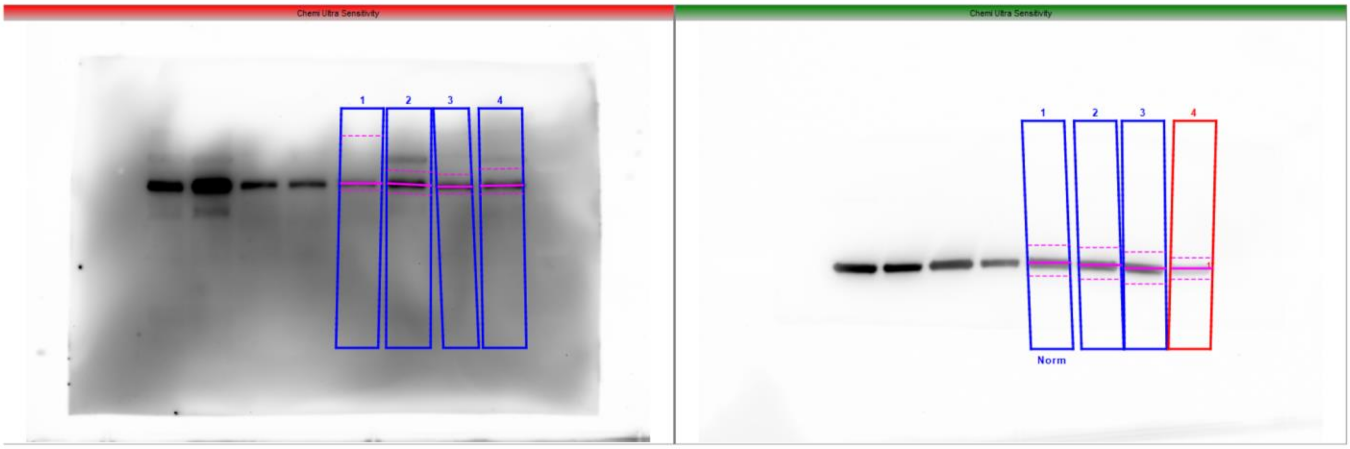

Lane	Band No.	Band Labe	Mol. Wt. (	Relative F	Adj. Volur	Volume (I	Abs. Quan	Rel. Quan	Band %	Lane %	Norm. Fac	Norm. Vol
1	1		N/A	0.312796	8365034	50225946	N/A	N/A	100	86.78305	1	8365034
1	1		N/A	0.621891	3775073	4847962	N/A	N/A	100	92.29719	1	3775073
2	1		N/A	0.317536	8545797	37250577	N/A	N/A	100	50.00445	1.021192	8726896
2	1		N/A	0.631841	3728638	4823542	N/A	N/A	100	93.09376	1.021192	3807654
3	1		N/A	0.327014	4918243	23514151	N/A	N/A	100	68.37814	1.028785	5059815
3	1		N/A	0.646766	3687790	4771520	N/A	N/A	100	92.75856	1.028785	3793943
4	1		N/A	0.322275	6611392	35044208	N/A	N/A	100	39.99568	2.906605	19216704
4	1		N/A	0.646766	1250711	2029931	N/A	N/A	100	88.88042	2.906605	3635322

5

Figure 6 TGF B R1

B-actin

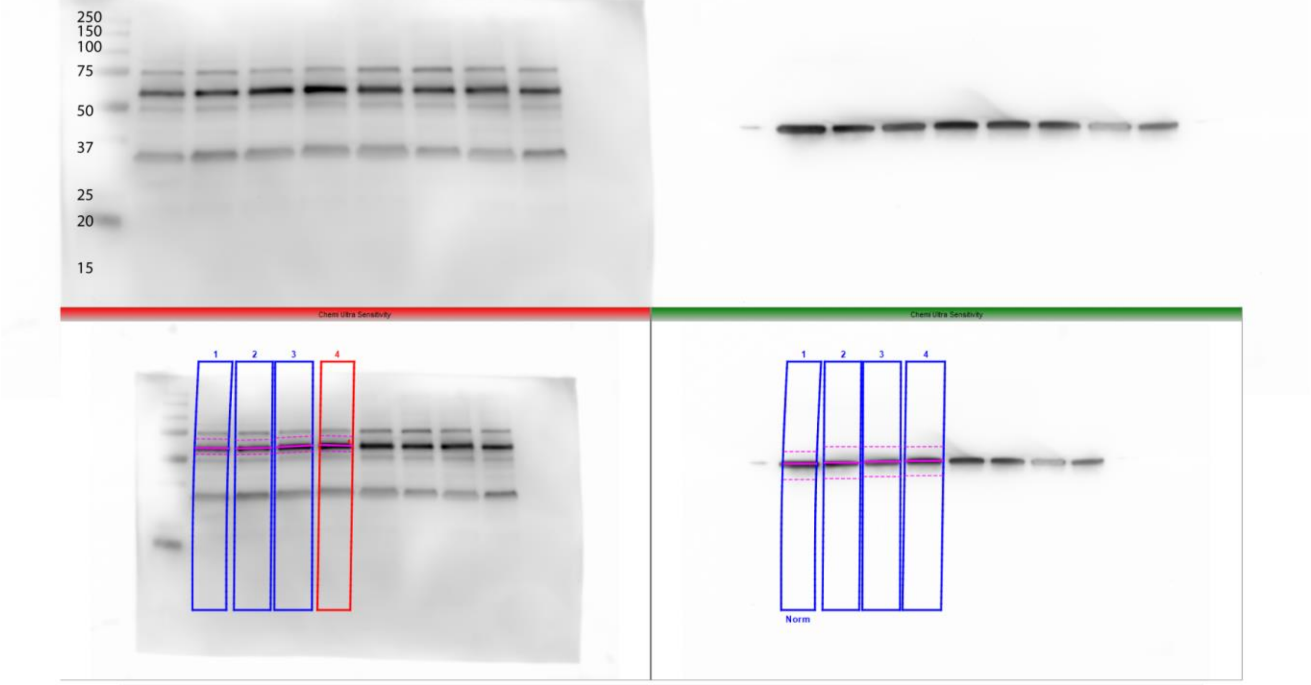

Lane	Band No.	Band Labe	Mol. Wt. (	Relative F	Adj. Volur	Volume (I	Abs. Quan	Rel. Quan	Band %	Lane %	Norm. Fac	Norm. Vol
1	1		N/A	0.348837	1769495	5913040	N/A	N/A	100	48.423	1	1769495
1	1		N/A	0.410853	19537070	21772380	N/A	N/A	100	92.67378	1	19537070
2	1		N/A	0.348837	2085706	6415426	N/A	N/A	100	42.55136	0.996595	2078604
2	1		N/A	0.406977	19995524	23078540	N/A	N/A	100	94.52551	0.996595	19927442
3	1		N/A	0.341085	2770482	7543770	N/A	N/A	100	56.4367	1.004595	2783212
3	1		N/A	0.403101	19323486	22032270	N/A	N/A	100	92.08185	1.004595	19412280
4	1		N/A	0.337209	2962590	7420704	N/A	N/A	100	59.67579	0.820076	2429547
4	1		N/A	0.399225	22392920	25024880	N/A	N/A	100	87.1088	0.820076	18363885

Figure 6 TGF B R2

B-actin

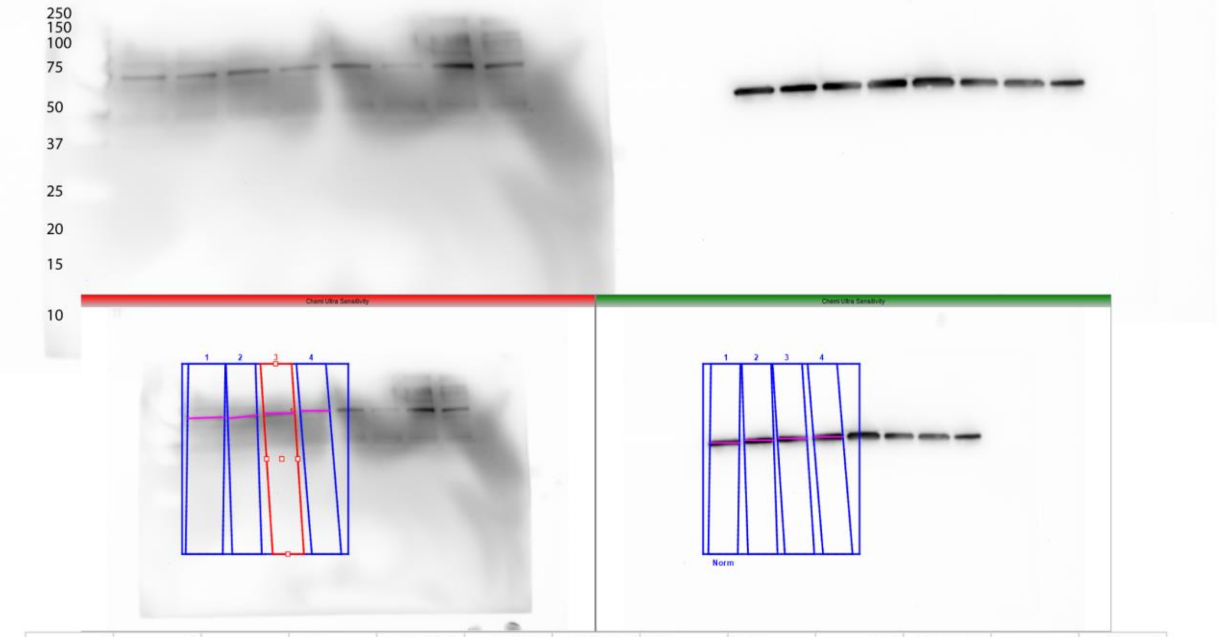

1	1		N/A	0.283105	504328	5409668	N/A	N/A	100	31.38382	1	504328
1	1		N/A	0.415525	2931810	3790850	N/A	N/A	100	94.87168	1	2931810
2	1		N/A	0.278539	412020	11548425	N/A	N/A	100	50.18545	0.827106	340784
2	1		N/A	0.401826	3544660	4425085	N/A	N/A	100	93.73849	0.827106	2931810
3	1		N/A	0.260274	620194	6686788	N/A	N/A	100	54.05179	1.001064	620853
3	1		N/A	0.392694	2928695	3734360	N/A	N/A	100	93.17736	1.001064	2931810
4	1		N/A	0.246575	457870	3757530	N/A	N/A	100	33.15508	0.866506	396747
4	1		N/A	0.383562	3383485	4219705	N/A	N/A	100	94.10568	0.866506	2931810
